# Supplementary material for: Micro-LED/van der Waals heterointegration for in-pixel processing display architecture
Source: Nat Commun. 2026 Feb 21;17:3049. doi: 10.1038/s41467-026-69786-2 (PMC13039472; doi:10.1038/s41467-026-69786-2)
Supplement: Supplementary file 1 — Supplementary Information [file 41467_2026_69786_MOESM1_ESM.pdf]

Supplementary Information

Micro-LED/van der Waals heterointegration for In-pixel processing display architecture

Fei Wang[1], Yuchun Wu[1], Hongling Chu[1], Jingbo Yang[1], Zhaorui Liu[1], Siqi Liu[1], Zhu Yang[1], Enlong Li[1], Jingjing Liu[1], Luqiao Yin[1], Mengjiao Li[1 *], Jianhua Zhang[1 *]

School of Microelectronics, Shanghai University, Jiading, Shanghai 201800, China;

[*]E-mail: mjli@shu.edu.cn; jhzhang@oa.shu.edu.cn

This Supplementary Information file includes:

Supplementary Notes 1 to 2

Supplementary Figures 1 to 24

Supplementary Tables 1 to 2

Supplementary References 1 to 30

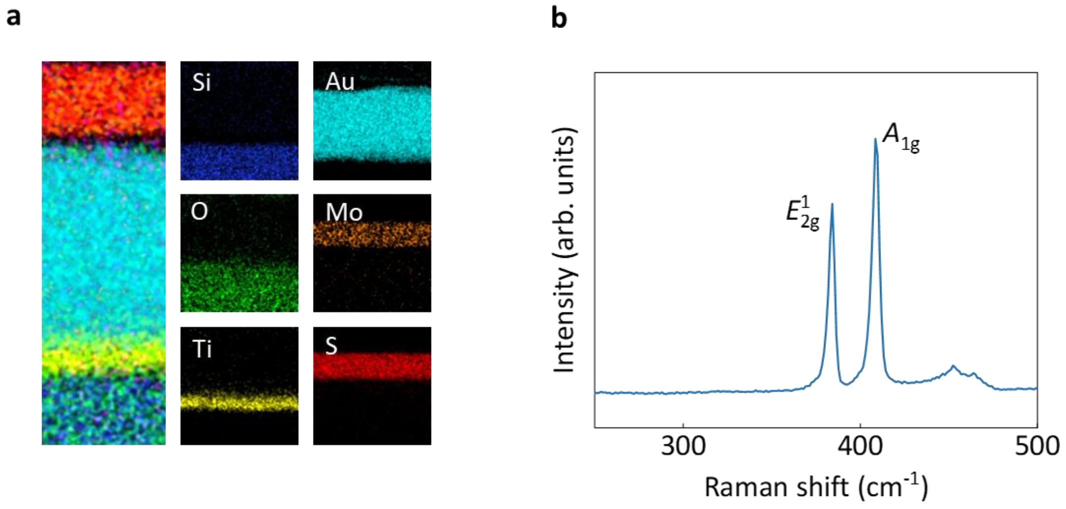

Supplementary Figure 1. Microscopic characterization of the MoS_2 driver transistors. a. The EDS mapping of S, Mo, Au, Ti, Si and O elements within the MoS_2 driver transistor. **b.** Raman spectra of the MoS_2 driver transistors. Characteristic vibrational peaks are observed at 383 and 408 cm^{-1}, corresponding to the vibration modes of E^1_{2g} and A_{1g}, respectively.

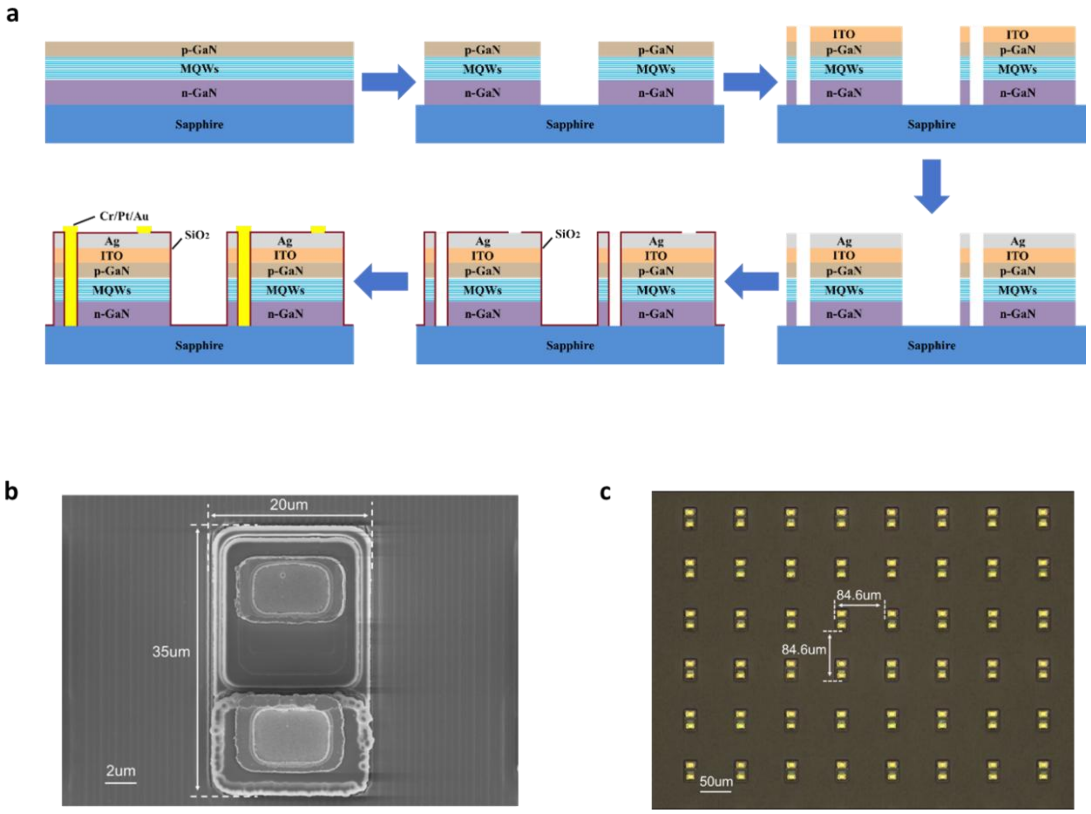

Supplementary Figure 2. Fabrication process and optical imaging of micro-LEDs. a. Schematic illustration of the micro-LED fabrication process. **b.** Optical micrograph of a single micro-LED device. **c.** Optical micrograph of a micro-LED array, illustrating the device layout and pitch.

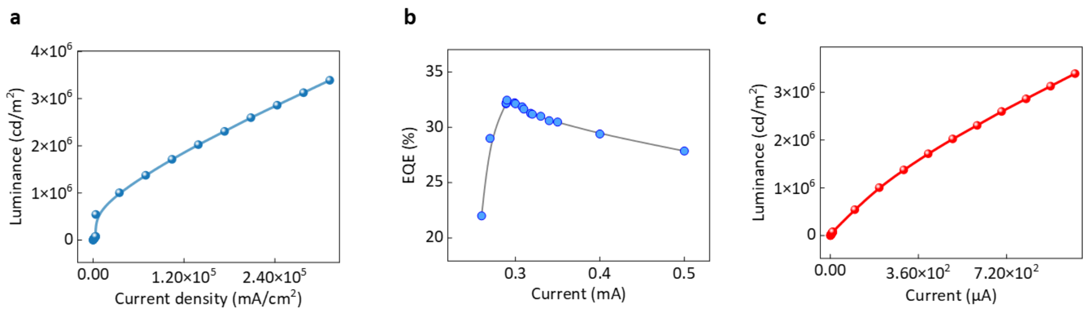

Supplementary Figure 3. Electroluminescence performance of the micro-LED. a. Relationship between the luminance and injection current of the micro-LED. **b.** External quantum efficiency (EQE) characteristics of the micro-LED. **c.** Relationship between the current and luminance of the micro-LED.

Supplementary Note 1. Micro-LED device fabrication and luminescence performance

This work employs a discrete flip-chip micro-LED process, which involves the following key steps (**Figure S2a**):

(i) Metal-organic chemical vapor deposition (MOCVD) was used to grow an n-type GaN layer (Si-doped), an InGaN/GaN multiple quantum well (MQW) light-emitting layer, and a p-type GaN layer (Mg-doped) sequentially on a sapphire substrate. Inductively coupled plasma (ICP) dry etching was then applied to remove the p-GaN and MQW layers, exposing the n-GaN layer.

(ii) An indium tin oxide (ITO) current-spreading layer was deposited on the GaN surface via sputtering, followed by ICP etching to pattern the ITO. An Ag reflective layer was subsequently formed by electron-beam evaporation to enhance top-side light extraction.

(iii) A SiO_2 isolation layer was deposited over the micro-LED structure by plasma-enhanced chemical vapor deposition (PECVD). Dry etching was performed to open vias in the SiO_2, exposing the underlying Ag reflector and n-GaN layer. Finally, patterned metal electrodes for individual pixels were formed by electron-beam evaporation on both the mesa and n-GaN regions.

Figure S2b-c presents a schematic of the fabricated discrete flip-chip micro-LED array and a scanning electron microscopy (SEM) image of a single pixel, clearly showing the pixel dimensions and pitch. In terms of current density, the device reaches a luminance of 3.39×10^6 cd/m^2 at a current density of 3.12×10^5 A/cm^2, demonstrating high-current injection capability and high luminance potential. Regarding external quantum efficiency (EQE), the device reaches a peak EQE of 32.46% at a current of 0.29 mA (**Figure S3**).

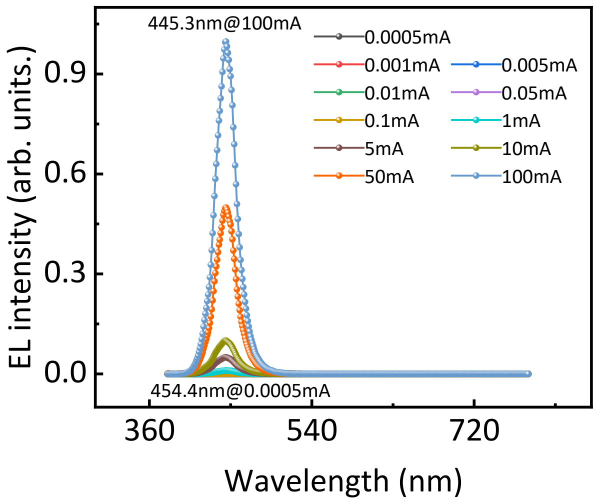

Supplementary Figure 4. Electroluminescence (EL) spectra of the IPPMLED at various current levels. The slight shift in the peak emission wavelength indicates the great emission purity across the tested current range.

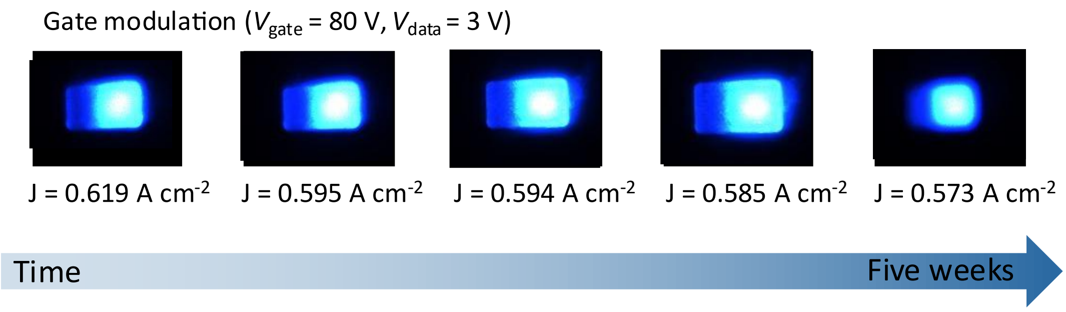

Supplementary Figure 5. Long-term luminance stability of IPPMLED device tested over a five-week period. The blue emission in the measured pixel is consistent, demonstrating stable performance throughout the test duration.

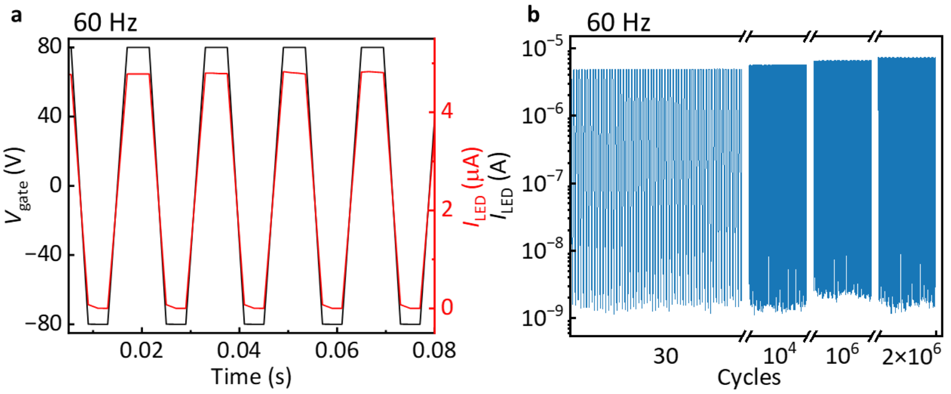

Supplementary Figure 6. The stable operation of the IPPMLED cell. (a) Stable switching behavior of the SiO_2-based IPPMLED under 60 Hz driving pulses at ±80 V. (b) Stable device performance maintained for over 2×10^6 switching cycles with a gate voltage swing of ±80 V.

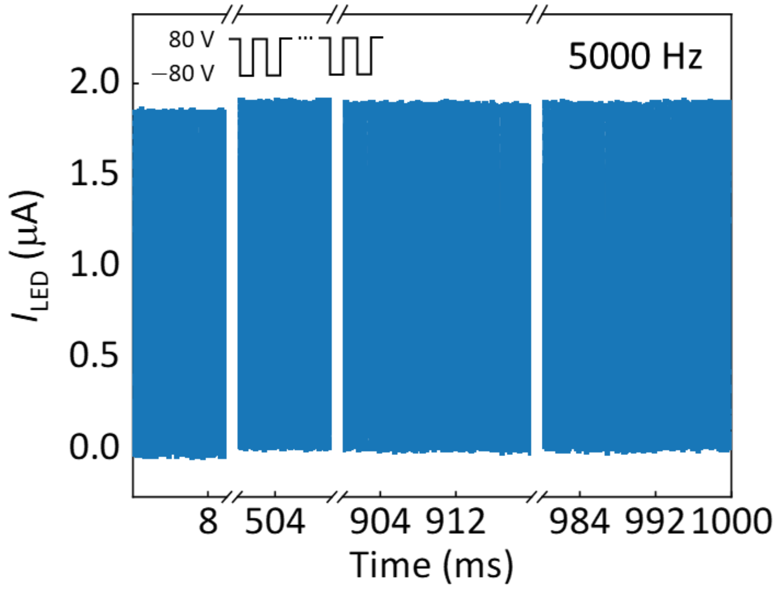

Supplementary Figure 7. Stable switching characteristics of the IPPMLED pixel at high frequency. The SiO_2-based IPPM LED pixels exhibit stable switching performance for over 10^3 cycles when driven at a high frequency of 5000 Hz with a gate voltage swing of ±80 V.

Supplementary Table 1 Benchmark performance survey of existing display technology.

Ref.	Display type	Display backplane	Assembly method	PPI	Luminance	Switching speed (Hz)	LED size
(*1*)	Micro-LED	(a-IGZO)TFT	Flip chip bonding	202.4	630 cd/m^2/5V	120	90×50 um^2
(*2*)	Micro-LED	CMOS	Flip chip bonding	169.5	4×10^6 cd/m^2/-	-	12×12 um^2
(*3*)	Micro-LED	CMOS	Flip chip bonding	33.87	250 cd/m^2/12uA	120	-
(*4*)	Micro-LED	CMOS	Wire bonding	2540	1×10^6 cd/m^2/-	-	6.5×6.5 um^2
(*5*)	Micro-LED	MoS_2	Monolithic Integration	508	2×10^4 cd/m^2/6V	60	10×10 um^2
(*6*)	Micro-LED	CMOS	COB	-	-	10^4	210 ×210 um^2
(*7*)	Micro-LED	CMOS	Micro transfer printing	1270	2190 cd/m^2/1.8V	-	30× 15 um^2
(*8*)	Micro-LED	CMOS	Vertical Non-alignment Bonding	3400	32987 cd/m^2/1.5A/cm^2	60	5×5 um^2
(*9*)	Micro-LED	PI	-	25.4	-	-	100×60 um^2
(*10*)	Micro-LED	ITO	Via Interconnect	1270	10737 cd/m^2/8V	-	10×10 um^2
(*11*)	Micro-LED	-	Wire bonding	907	11800 cd/m^2/-	-	20×20 um^2
(*12*)	Micro-LED	CMOS	Flip chip bonding	253.9	1×10^6 cd/m^2/8.3V	100	80×80 um^2
(*13*)	QLED	ITO	Via Interconnect	1278	-	-	10×10 um^2
(*14*)	QLED	ITO	Via Interconnect	16	250 cd/m^2/7V	-	1×4 mm^2
(*15*)	QLED	MoS_2	Via Interconnect	-	5137 cd/m^2/7V	-	100×100 um^2
(*16*)	QLED	ITO	Via Interconnect	-	70,650 cd/m^2/10.5V	-	-
(*17*)	OLED	TFT	Via Interconnect	2000	1000 cd/m^2/2.6V	-	4.2×4.2 um^2
(*18*)	OLED	ITO	Via Interconnect	-	14,700 cd/m^2/2.4V	-	-
(*19*)	OLED	TFT (Carbon Nanotube)	Via Interconnect	52.8	10,000 cd/m^2/12V	-	480×480 um^2
(*20*)	OLED	MoS_2	Via Interconnect	53.12	791cd/m^2/9V	-	-
This work	Micro-LED	MoS_2	Flip chip bonding	300	$3\times10^5$$cd/m^2$/8V	5×10^3	20×35um^2

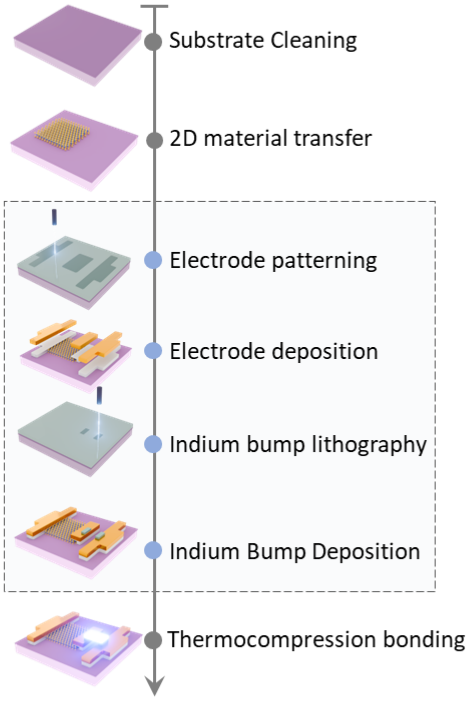

Supplementary Figure 8. Schematic illustration of the fabrication process for top-contact MoS_2 transistors. Key steps include the transfer of MoS_2 material followed by patterned electrode fabrication.

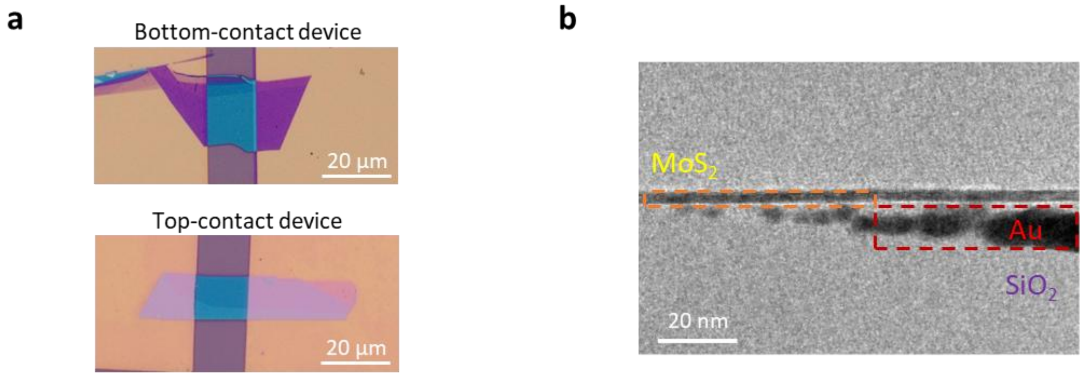

Supplementary Figure 9. Structural characterization of bottom-contact and top-contact driver transistors. a. Optical microscope images of the bottom-contact and top-contact MoS_2 transistors. **b**. Cross-sectional TEM image that confirms the local structure of the bottom-contact MoS_2 transistors.

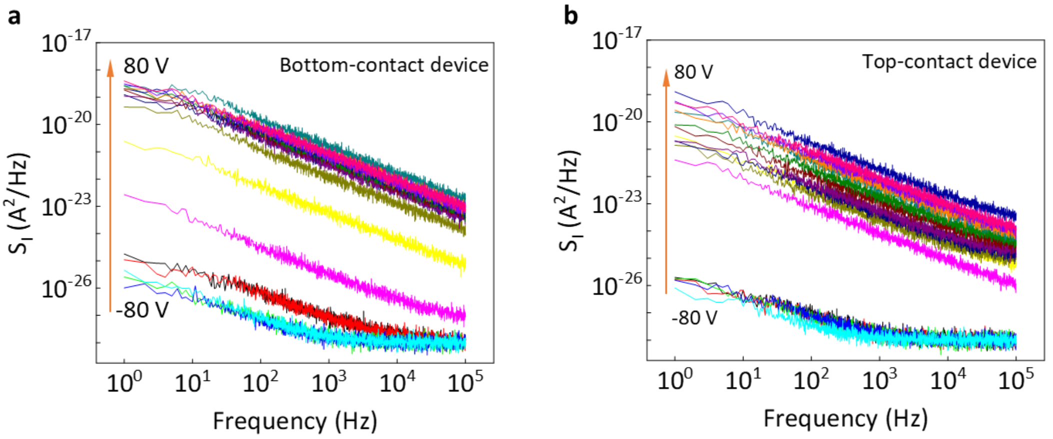

Supplementary Figure 10. Low-frequency noise characteristics of MoS_2 transistors with different contact configurations. a. Low-frequency noise characteristics of bottom-contact MoS_2 transistors. **b.** Low-frequency noise characteristics of top-contact MoS_2 transistors.

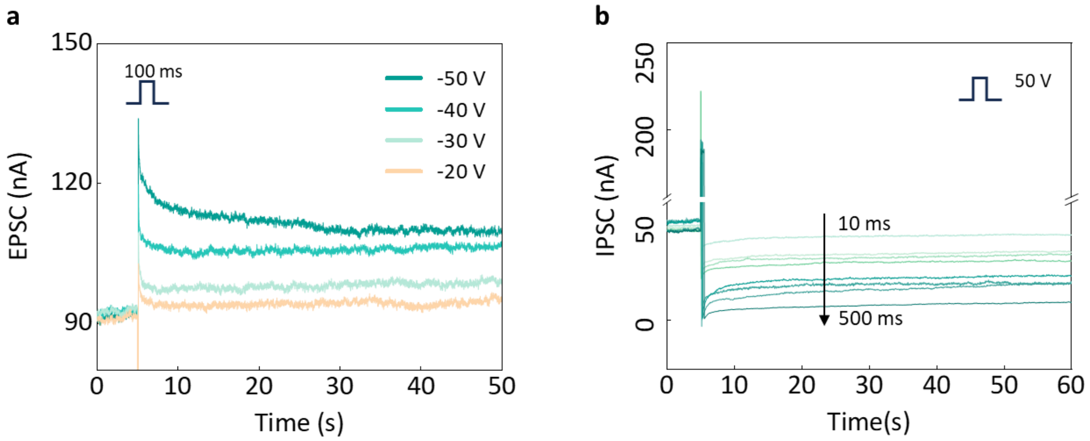

Supplementary Figure 11. Postsynaptic current behavior of the IPPMLED under different pulse conditions. a. EPSC characteristics of the IPPMLED under different pulse amplitudes, measured with a 100 ms pulse width. **b.** IPSC characteristics of the IPPMLED under different pulse widths.

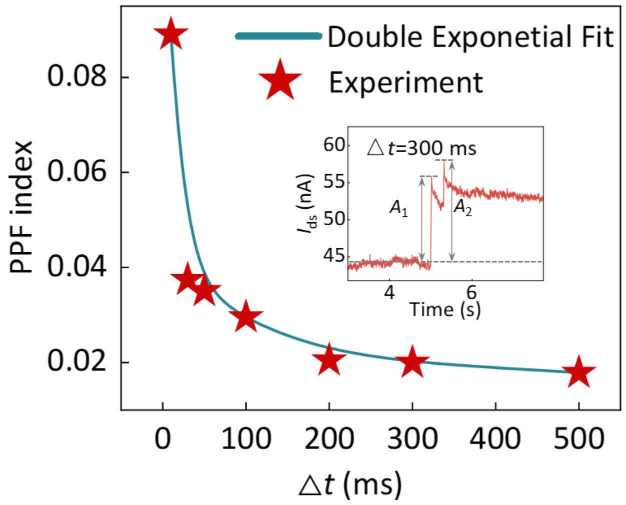

Supplementary Figure 12. PPF index as a function of the interval time between paired negative gate pulses. The solid line represents the double exponential fit result. The inset shows the current response to a pair of pulses. The PPF index is defined as the ratio of the amplitude difference after paired spikes (A_2-A_1) and a current change of the first spike (A_1).

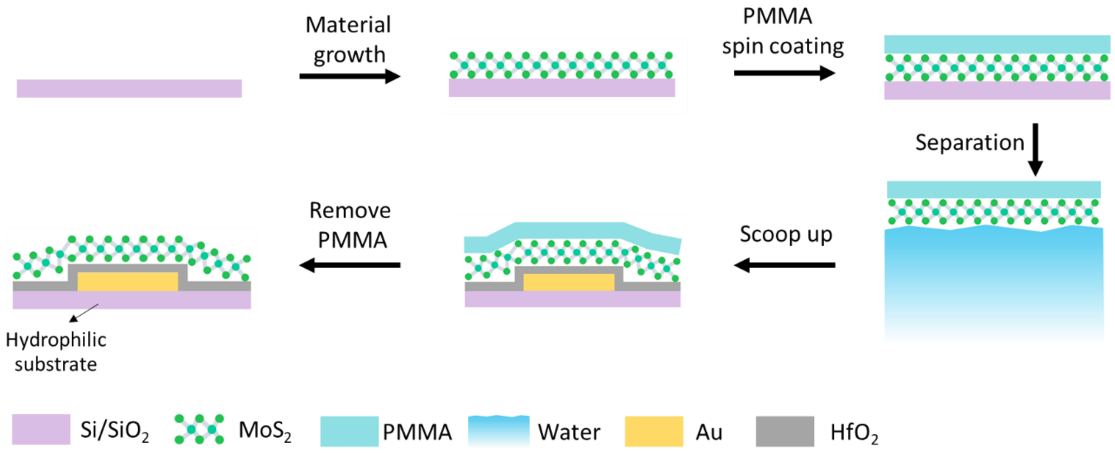

Supplementary Figure 13. Schematic diagram of the PMMA-assisted wet transfer process for large-area two-dimensional materials. This schematic illustrates the hydrophilic treatment of the substrate and the mild chemical etching process used to remove the PMMA support layer.

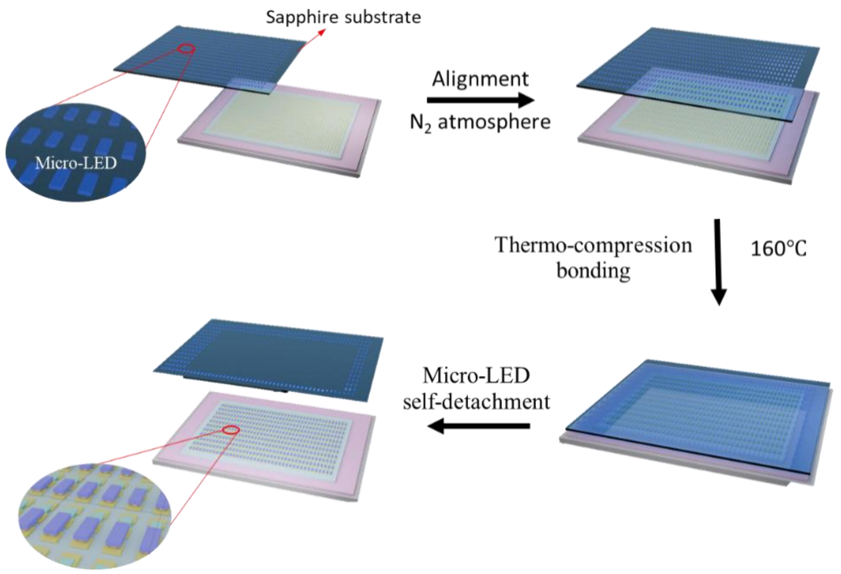

Supplementary Figure 14. Schematic diagram of the micro-LED low-temperature thermo-compression bonding process. The procedure is conducted in an N_2 atmosphere to ensure device performance is maintained.

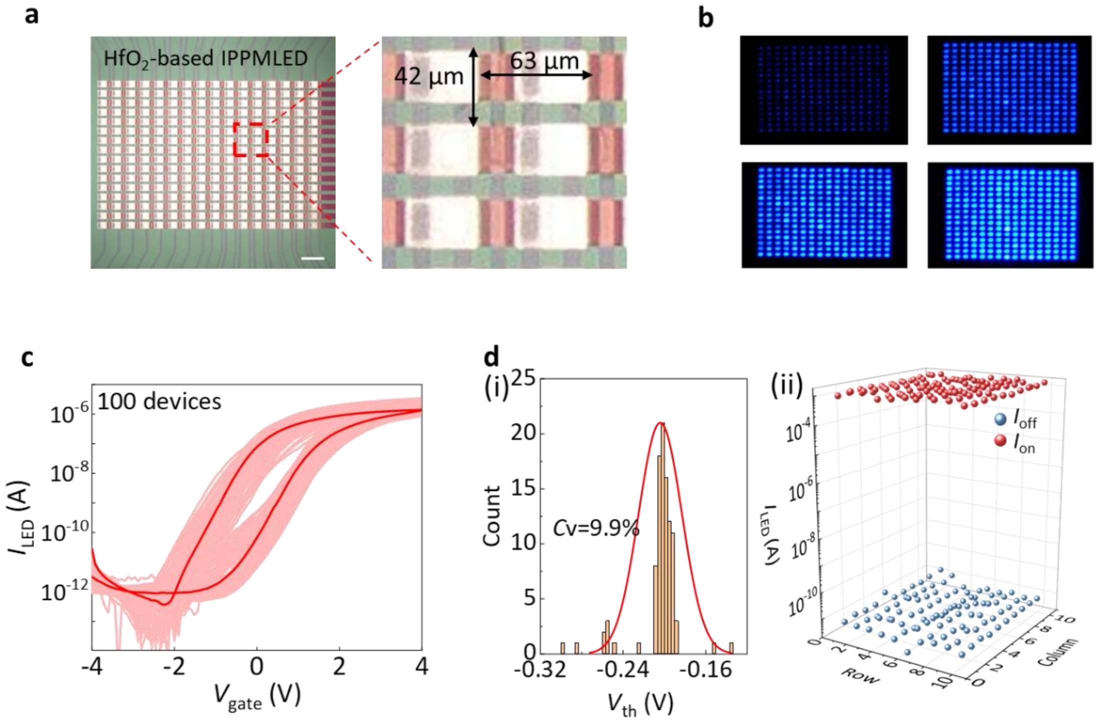

Supplementary Figure 15. Uniformity characterization of HfO_2-based IPPMLED arrays. a. An overview optical micrograph (scale bar: 100 μm) and a high-magnification image of the fabricated HfO_2-based IPPMLED array. **b**. Uniform light emission from the operational 16×16 array, demonstrating four distinct luminance levels. **c.** Transfer characteristic curves of 100 individual HfO_2-based IPPMLED devices at a data voltage of 3 V. **d.** Statistical distributions of the V_{th} (i) and on/off state (ii) extracted from the transfer curves.

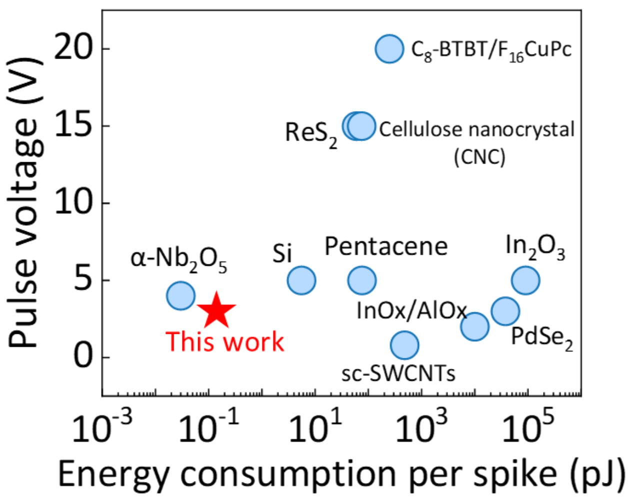

Supplementary Figure 16 Benchmarking of energy consumption per spike across various emerging synaptic electronic devices with different channel materials[21-30], showing the energy consumption advantage of vdW-driven transistors based on HfO_2 dielectric layers.

Supplementary Table 2 The surveyed studies about the energy consumption of various micro-LED technologies.

References	Display type	Driver transistor	V_{data}	V_{gate}	Energy consumption
Nat. Nanotechnol. 16, 1231-1236 (2021)	Micro-LED	MoS_2 driver transisitor	8 V	8 V	12.8 mW
Displays 87, 102997 (2025).	Micro-LED	Si-CMOS	4 V	4.5 V	12 mW
Adv. Mater. 37, 2416015 (2025) .	Micro-LED	Poly-Si transisitor	-4 V	-10 V	36 µW
Adv. Mater. 37, 2411999 (2025) .	Micro-LED	/	2.8 V	/	> 150 mW cm^{-2}
Nano Energy 135, 110613 (2025).	Micro-LED	/	6 V	/	0.9 mW (60 A/cm^2)
Nat. Commun. 16, 9612 (2025) .	Micro-LED	/	3.3 V	/	864.6 uW
Adv. Optical Mater. 13, 2500271 (2025)	Micro-LED	/	4.3 V	6 V	42.23 W /cm^2 (222 A/cm^2)
Nat. Nanotechnol. 175, 500-506 (2022).	Micro-LED	MoS_2 transisitor	8 V	8 V	21 mW (20 A/cm^2)
Light Sci. Appl. 12, 258 (2023)	Micro-LED	Si-CMOS	3.2 V	5 V	7.04 uW
This work	Micro-LED	MoS_2 transisitor	3 V	3 V	14.4 µW (95.1 A/cm^2)

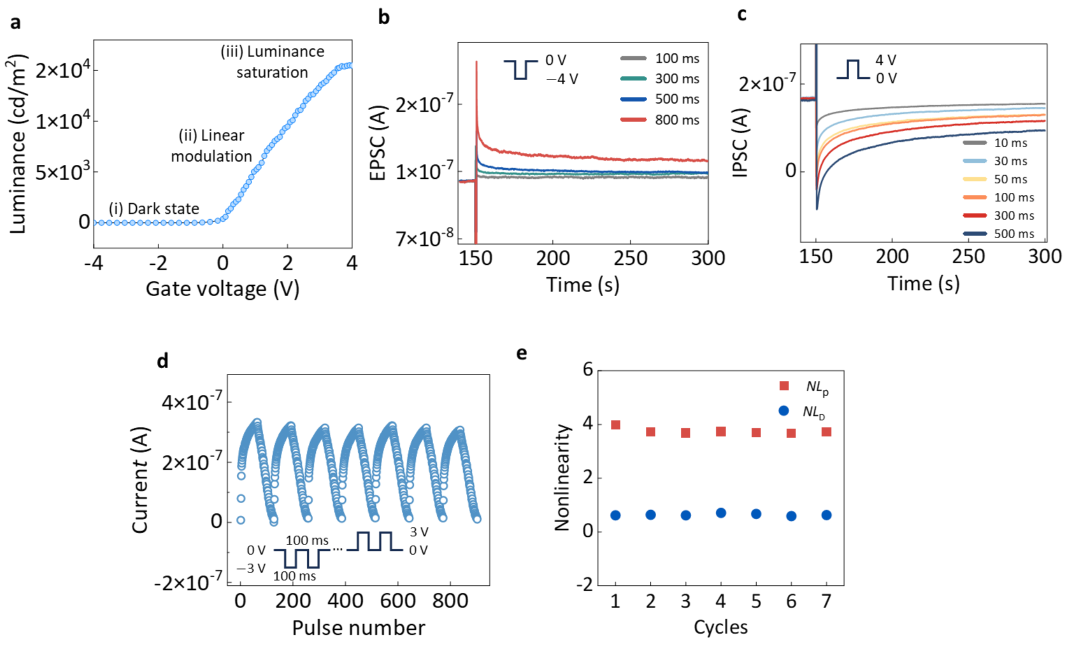

Supplementary Figure 17. Voltage-dependent luminescence and memory characteristics of the HfO_2-based IPPMLED. a. Gate voltage–dependent luminance characteristics and the corresponding light emission behaviors of IPPMLED devices under different stages. b. EPSC characteristics of the IPPMLED under varying pulse amplitudes at negative gate voltages. c. IPSC characteristics of the IPPMLED under varying pulse amplitudes at positive gate voltages. **d.** Potentiation-depression characteristics of the IPPMLED device. **e.** Nonlinearity of potentiation (NL_P) and depression (NL_D), obtained from the experimental data in (d).

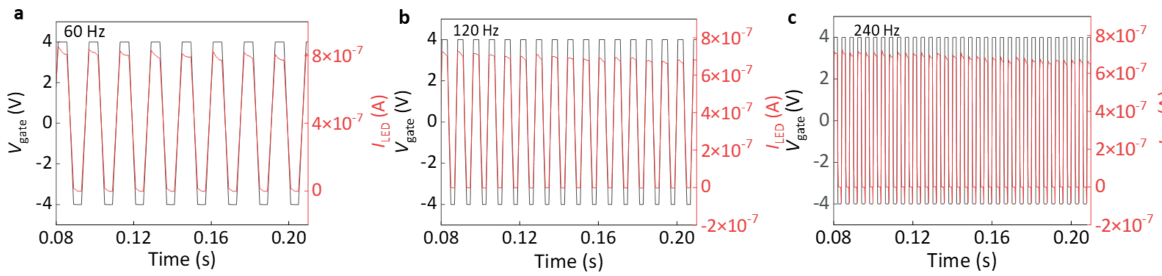

Supplementary Figure 18. Current response of the IPPMLED under different pulse frequencies. Current response characteristics of the IPPMLED at pulse frequencies of 60 Hz (a), 120 Hz (b) and 240 Hz (c), demonstrating the rapid refreshing potential of the proposed pixel driving design.

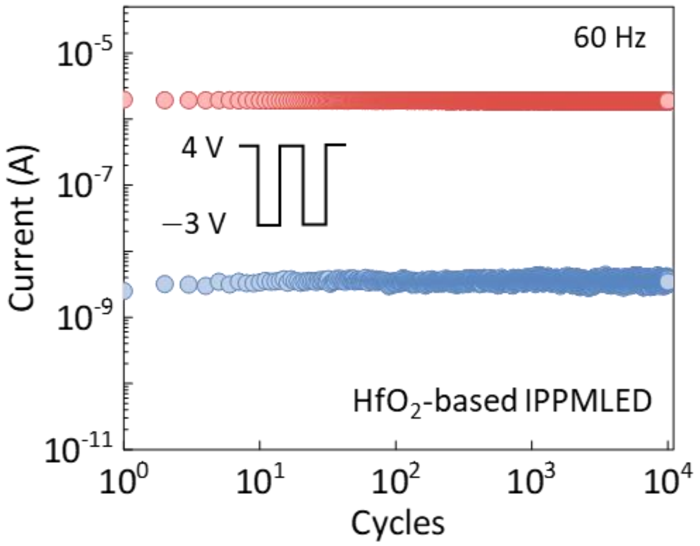

Supplementary Figure 19. Endurance characteristics of the HfO_2-based IPPMLEDs.
The endurance characteristics of HfO_2-based IPPMLED over 10^4 cycles.

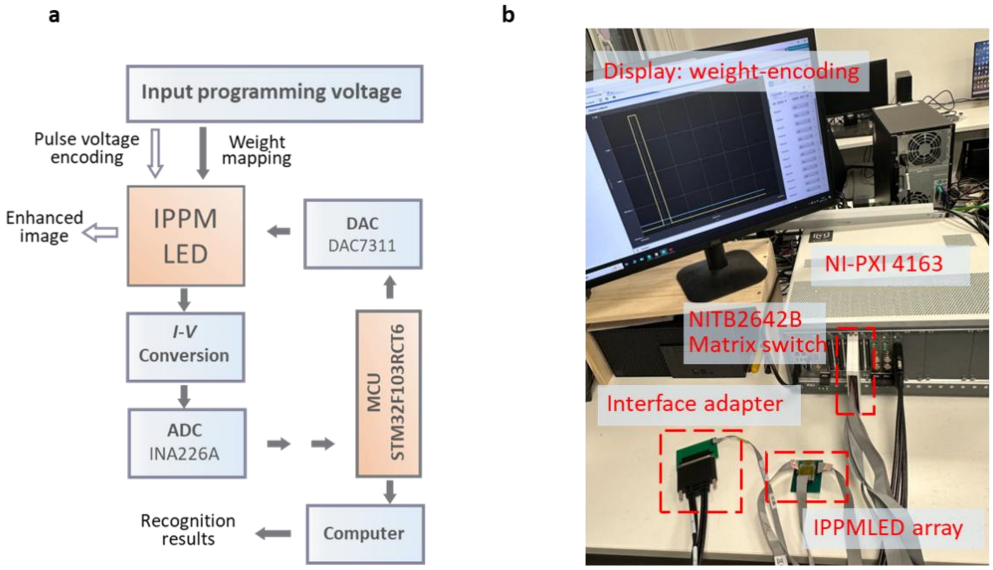

Supplementary Figure 20. Demonstration of the in-pixel display processing based on IPPMLED arrays. a. Flowchart of the weight mapping and inference process, incorporating the HfO_2-based IPPMLED array and peripheral electronics**. b.** Photograph of the display processing platform, showing the matrix-configured IPPMLED array mounted on a custom PCB for multi-channel measurement.

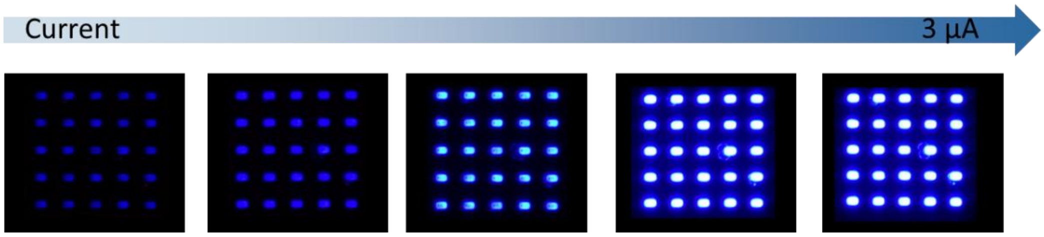

Supplementary Figure 21. Current-dependent luminance response of a 5×5 IPPMLED pixel array. Luminance gradually increases as the driving current rises from 0 to 3 μA, demonstrating uniform and programmable pixel response for high-fidelity display control.

Supplementary Note 2. Contrast stretching for display enhancement.

For contrast stretching in image processing, the algorithm determines how the pixel values of the image are adjusted. The piecewise linear contrast stretching method is characterized by its ability to perform differentiated mappings across discrete grayscale intervals, thereby enabling more flexible enhancement of local image contrast. As shown in Equation 1, the mapping curve is a straight line with a slope greater than 1, clipped at 0 and 255:

$$P\text{out}=\begin{cases} 0, & P_{\text{in}}<P_{\text{min}} \\ \alpha(P_{\text{in}}-\mu)+\mu, & P_{\text{min}}<P_{\text{in}}<P_{\text{max}} \\ 255, & P_{\text{in}}>P_{\text{max}} \end{cases} \tag{1}$$

where P_{in} and P_{out} are the input pixel value and output pixel value, respectively, and α is the contrast adjustment factor (α > 1 enhances contrast, 0 < α < 1 reduces it). The term μ refers to the mean pixel intensity of the entire image. The threshold P_{min} defines the weak-interference pixel range, where values of P_{in} below P_{min} are mapped to 0 in the output. Similarly, P_{max} marks the strong-feature pixel threshold, where values of P_{in} above P_{max} are stretched to the maximum output value of 255. This process physically widens narrow-band pixel distributions via pixel remapping.

Based on the well-characterized piecewise voltage-luminance response of our IPPMLED (Figure S17a), we implemented device-level piecewise linear pixel remapping via voltage-modulated luminance (Equation 2):

$$\text{B}(V)=\begin{cases} 0, & V<V_{\text{min}} \\ \beta V_{\text{in}}+\gamma, & V_{\text{min}}<V<V_{\text{max}} \\ 2\times10^{4}, & V>V_{\text{max}} \end{cases} \tag{2}$$

where V_{in} is the regulation voltage, β is the regulation factor, and γ is the regulation constant.

Therefore, a direct mapping between the voltage-luminance response and contrast stretching algorithm encodes input image pixel values into corresponding gate voltages, redistributing clustered luminance levels across a broader range and generating enhanced images displayed in real-time.

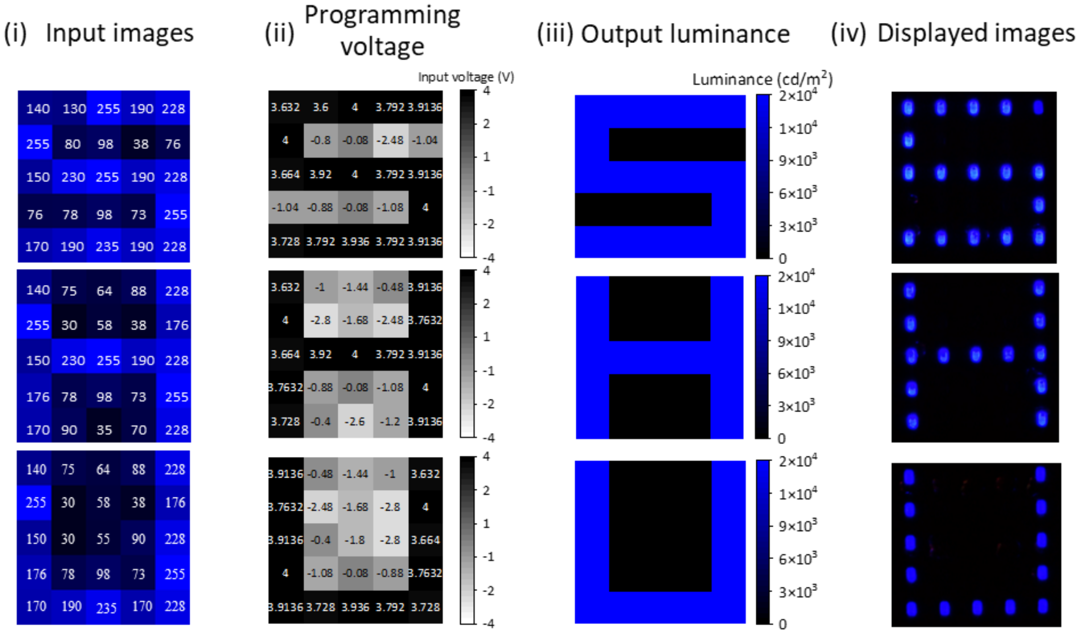

Supplementary Figure 22. Image enhancement workflow using the IPPMLED array. It includes the noise-contaminated input images of the characters S, H, and U (i), the corresponding programming voltages (ii), the luminance responses (iii), and the enhanced images reconstructed by the IPPMLED array (iv).

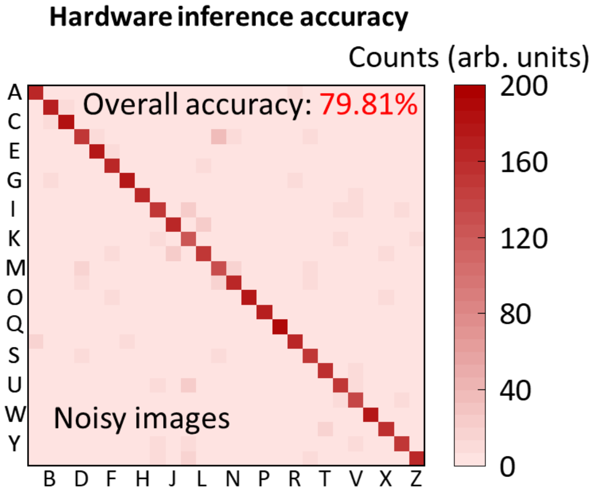

Supplementary Figure 23. Confusion matrix of recognition results for original noisy alphabetic images.

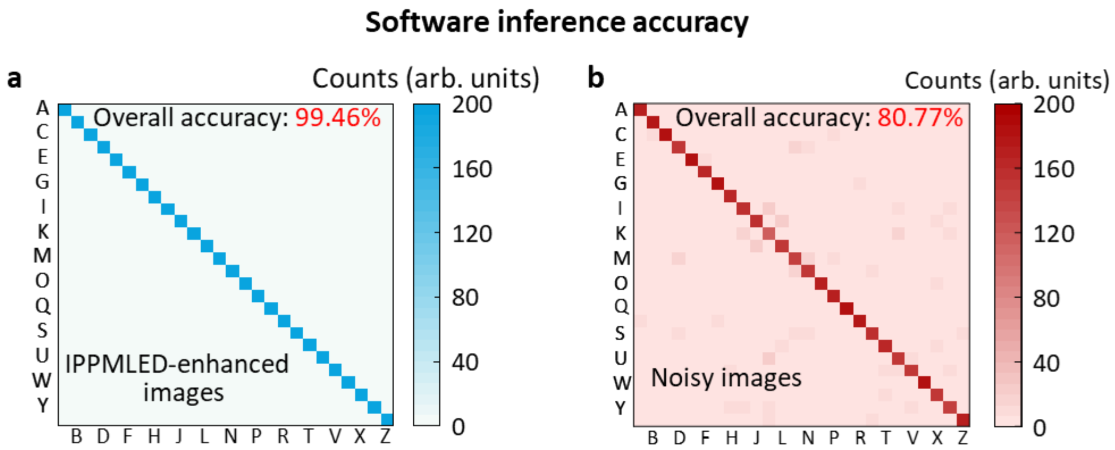

Supplementary Figure 24. Confusion matrices of software inference accuracy. a,b. Software inference results on IPPMLED-enhanced images (a) and noisy images (b).

Supplementary References

1. Um, J. G. et al. Active-matrix GaN μ-LED display using oxide thin-film transistor backplane and flip chip LED bonding. *Adv. Electron. Mater.* 5, 1800617 (2019).
2. Day, J. et al. III-nitride full-scale high-resolution microdisplays. *Appl. Phys. Lett.* 99, 031116 (2011).
3. Ahn, H. -A., Hong, S. -K. &Kwon, O. -K. An active matrix micro-pixelated LED display driver for high luminance uniformity using resistance mismatch compensation method. *IEEE T CIRCUITS-II* 65, 724–728 (2018).
4. Templier, F. GaN-based emissive microdisplays: A very promising technology for compact, ultra-high brightness display systems. *J. Soc. Inf. Disp.* 24, 669–675 (2016).
5. Hwangbo, S. et al. Wafer-scale monolithic integration of full-colour micro-LED display using MoS_2 transistor. *Nat. Nanotechnol.* 17, 500–506 (2022).
6. Peng, D., Zhang, K. & Liu, Z. Design and fabrication of fine-pitch pixelated-addressed micro-LED arrays on printed circuit board for display and communication applications. *IEEE J. Electron Devices Soc.* 5, 90–94 (2017).
7. Cok, R. S. et al. Inorganic light-emitting diode displays using micro-transfer printing. *J. Soc. Inf. Disp.* 25, 589–609 (2017).
8. Wu, H. et al. Ultra-high brightness micro-LEDs with wafer-scale uniform GaN-on-silicon epilayers. *Light Sci. Appl.* 13, 284 (2024).
9. Shim, H. C. et al. Full-color micro-LED display with photo-patternable and highly ambient-stable perovskite quantum dot/siloxane composite as color conversion layers. *Sci. Rep.* 13, 4836 (2023).
10. An, H. J., Kim, M. S. & Myoung, J. -M. Strategy for the fabrication of perovskite-based green micro LED for ultra high-resolution displays by micro-molding process and surface passivation. *Chem. Eng. J.* 453, 139927 (2023).
11. Xiao, Y. et al. A 64 × 64 GaN micro LED monolithic display array: Fabrication and light crosstalk analysis. *Micromachines* 16, 207 (2025).
12. Herrnsdorf, J. et al. Active-matrix GaN micro light-emitting diode display with unprecedented brightness. *IEEE Trans. Electron Devices.* 62, 1918–1925 (2015).
13. Li, X. et al. CdSe/ZnS quantum dot patterned arrays for full-color light-emitting diodes in active-matrix QLED display. *ACS Appl. Nano Mater.* 7, 9086–9094

(2024).

14. Lai, K. Y. et al. Patterned-bank-free electroluminescent quantum dot emitting array for passive-matrix QLED display. *Adv. Mater. Technol.* 7, 2100889 (2022).

15. Ji, S. et al. Perovskite Light-Emitting Diode Display Based on MoS_2 Backplane Thin-Film Transistors. *Adv. Mater.* 36, 2309531 (2024).

16. Yang, Z. et al. All-solution processed inverted green quantum dot light-emitting diodes with concurrent high efficiency and long lifetime. *Mater. Horiz.* 6, 2009–2015 (2019).

17. Choi, S. et al. Thin-film transistor-driven vertically stacked full-color organic light-emitting diodes for high-resolution active-matrix displays. *Nat. Commun.* 11, 2732 (2020).

18. Fröbel, M. et al. Three-terminal RGB full-color OLED pixels for ultrahigh density displays. *Sci. Rep.* 8, 9684 (2018).

19. Zou, J. et al. Carbon nanotube driver circuit for 6 × 6 organic light emitting diode display. *Sci. Rep.* 5, 11755 (2015).

20. Choi, M. et al. Full-color active-matrix organic light-emitting diode display on human skin based on a large-area MoS_2 backplane. *Sci. Adv.* 6, eabb5898 (2020).

21. Xu, H. et al. A low-power vertical dual-gate neurotransistor with short-term memory for high energy-efficient neuromorphic computing. *Nat. Commun.* 14, 6385 (2023).

22. Seo, S. et al. The gate injection-based field-effect synapse transistor with linear conductance update for online training. *Nat. Commun.* 13, 6431 (2022).

23. Li, L. et al. Floating-gate photosensitive synaptic transistors with tunable functions for neuromorphic computing. *Science China Materials.* 64, 1219–1229 (2021).

24. Xie, T. et al. Carbon nanotube optoelectronic synapse transistor arrays with ultra-low power consumption for stretchable neuromorphic vision systems. *Adv. Funct. Mater.* 33, 2303970 (2023).

25. Liang, X. C. et al. Multimode transistors and neural networks based on ion-dynamic capacitance. *Nat. Electron.* 5. 859–869 (2022).

26. Ma, M. X. et al. Multiplexed neurochemical transmission emulated using a dual-excitatory synaptic transistor. *npj 2D Mater. Appl.* 5, 23 (2021).

27. Li, F. F. et al. An artificial visual neuron with multiplexed rate and time-to-first-

spike coding. *Nat. Commun.* 15, 3689 (2024).

28. Chen, Y. B. et al. All two-dimensional integration-type optoelectronic synapse mimicking visual attention mechanism for multi-target recognition. *Adv. Funct. Mater.* 33, 2209781 (2023).

29. Han, M. J. & Tsukruk, V. V. Trainable bilingual synaptic functions in bio-enabled synaptic transistors. *ACS Nano* 17, 18883–18892 (2023).

30. Hao, Z. Q. et al. Retina-inspired self-powered artificial optoelectronic synapses with selective detection in organic asymmetric heterojunctions. *Adv. Sci.* 9, 2103494 (2022).
